# Dental Erosion and Dentinal Sensitivity amongst Professional Wine Tasters in South East Queensland, Australia

**DOI:** 10.1155/2014/516975

**Published:** 2014-01-16

**Authors:** Roy George, Allison Chell, Belinda Chen, Rebecca Undery, Humza Ahmed

**Affiliations:** School of Dentistry and Oral Health, Griffith University, Gold Coast, QLD 4215, Australia

## Abstract

*Background/Aims*. Professional wine tasters face a hidden occupational hazard due to the high acid content in wine. This study evaluates the self-perceived dentinal sensitivity and erosive effects of wine on the professional wine tasters of the Granite Belt and the Scenic Rim regions of South East Queensland, Australia. *Methods*. Seventy wineries were contacted and participants were surveyed about their professional wine tasting experience and oral health. Participants were also required to rate their tooth sensitivity prior to being examined for erosion using a modified Smith & Knight tooth wear index. The data were analysed using Mann Whitney *U* test and Spearman's correlation test. *Results*. The results showed that most participants (25 males, 22–66 yrs), brushed twice a day; however, the majority did not floss daily and had limited knowledge of the erosive effect of wine. There was a direct correlation between years of wine tasting, age of participants, and the erosion index. Correlation was not observed between the participant's sensitivity index and erosion index. *Conclusion*. The lack of significant experience of dentinal hypersensitivity amongst professional wine tasters should not prevent oral health practitioners from providing necessary counselling and undertaking preventive measures, as tooth wear can have serious long-term effect on oral health of an individual.

## 1. Introduction 

There is a hidden occupational hazard that faces many professional wine tasters worldwide. The acidity of wine on the permanent dentition can be quite detrimental with an increased risk of erosion of dental hard tissue [1]. Dental erosion is defined as a superficial loss of tooth substance by a chemical process that does not involve bacteria [2]. Dental erosion can result from numerous causes; however, extrinsic dietary factors are amongst the most common etiological factors. Dental erosion can present initially as a smooth, silky-shining glazed enamel surface or, in more advanced cases, as shallow concavities and/or rounding and grooving of the cusps at the margins [3]. The demineralising effect of acid in wine can also increase the susceptibility of tooth or teeth to mechanical abrasion, such as tooth brushing [4].

In Australia, an average person drinks 23.19 litres of wine a year, which has increased over the past ten years [5]. Wine is frequently consumed in Australia as a way of life, being closely associated with both business and leisure. Over the last two hundred years, the Australian wine industry has grown immensely from only a few small plantations to a world-renowned industry, known for its quality and value. Australia has approximately 2,000 wine companies and the sector employs an estimated number of 31,000 people [6]. In 2006, Australia was ranked the 6th largest wine producer in the world and has remained amongst the top 10 wine producing nations for many years [6]. In 2007, Australia had a record 3 billion dollars' worth of wine export [6]. In 2011, the Wine Export Approval Report (WEAR) showed that Australian exports earned A $1.96 billion [7].

In Queensland, the Granite Belt region is the premier wine region with over 45 wineries [8]. The Granite Belt in South East Queensland is centred in the town of Stanthorpe (Queensland Wine Industry Association 2008). The other major area around this belt that contributes significantly to wine production is the Scenic Rim region. The Scenic Rim region encompasses Mt Tamborine and the Countrysides of Canungra, Harrisville, Mt Alford, Boonah, Beaudesert, Rathdowney, Kooralbyn, Kalbar, Aratula, Harrisville, and Peak Crossing [9, 10].

The critical point at which enamel dissolves is reported to be between 5.0 and 5.7 pH. The acidity of wine (pH 3.0 to 3.8) indicates that long-term exposure of the teeth can result in serious tooth erosion [11]. Lussi and Jaeggi reported that full-time wine tasters taste on average 20–50 different wines, working nearly 5 days a week in Sweden [12]. Wine tasters are very susceptible to the negative effects of wine on oral health [11]. During wine tasting, wine is sipped, swirled, or swished around the mouth for approximately 30–60 seconds, increasing the risk of enamel and dentine erosion [13]. Mandel reported that wine tasters are very susceptible to acid erosion due to the frequency and longevity of wine exposure to the enamel surfaces [11]. The dental health effects of a typical adult suffering long-term dental erosion include staining, demineralised erosive lesions, dentinal sensitivity and exposure of dentine or in very severe cases may lead to irreversible pulpitis and necrosis of the pulp [3, 11, 14, 15]. The beneficial effects of saliva with its buffering capacity and its ability to form a protective enamel pellicle are diminished by the acidity of wine [16].

This study was done to evaluate the self-perceived dentinal sensitivity and erosive effect amongst professional wine tasters in the Granite Belt and Scenic Rim regions of South East Queensland, Australia.

## 2. Methods

The human ethical committee, Griffith University, Australia, approved this study. Professional wine tasters in (including wine judges and wine makers) Granite Belt and Scenic Rim region were approached to participate. In all, 70 wineries were contacted (45 in Granite Belt and 25 in Scenic Rim region). Participants were provided with an information sheet describing the current research and were then required to sign a consent form. Consenting participants were required to complete an anonymous health survey prior to being examined orally. The first part of the survey required participants to provide personal information such as age and gender. The second part of the survey required the participants to provide information on (1) the number of years as a professional wine taster, (2) number of sips taken for tasting on an average working day, (3) number of days of tasting per month, (4) type of wine tasted, (5) oral hygiene maintenance (brushing frequency, flossing frequency, brushing immediately before or after wine tasting, and use of additional oral health products), (6) personal opinion on detrimental effects of wine tasting (open ended question), and (7) finally participants were asked to rate their tooth sensitivity. Participants rated their sensitivity on a Liker's Scale of 1 to 5, where 1 was not sensitive and 5 was extremely sensitive.

The third part of the study involved oral examination of the participants for the level of erosion present using the modified Smith & Knight tooth wear index [17]. The index records incisal/cuspal wear for groups of teeth by identifying tooth loss on a five-point scale ranging from 0 to 5. For each tooth, a score is given based on a single feature, which indicated the highest score. Three separate scoring definitions were specified, namely, for incisors, canines, and multicusp teeth (premolars and molars). Calibrated dental science students conducted all oral examination to evaluate erosion index. The data was analysed with SPSS (*IBM SPSS Version 21)* statistical software using Mann Whitney *U* test and Spearman's correlation test.

## 3. Results 

Of the 70 wineries contacted only 25 professional wine tasters consented to participate in this study. The primary reason for low participating numbers was due to the region being a new and upcoming wine belt region and the fact that many worked in more than one winery. All participants completed the survey questionnaire and participated in the on site oral examination to evaluate individual erosion index. All participants in this study were males and were in the age group of 22–66 yrs. The work experience of the participants ranged from 1 to 40 years, with an average age of 27.90 (±9.50) as the age of starting out in the profession. The majority of the participants brushed their teeth two times or more a day (68%); however, only 40% of the participants flossed daily and only 40% used any additional oral health products (e.g., mouth rinse and fluoride gels). It is interesting to note that none of the participants brushed their teeth immediately before or after wine tasting. Participant's awareness of the detrimental effects of wine tasting was low, with only 11 participants (44%) aware of the effects of wine on erosion of teeth.

There was a significant correlation between years of wine tasting and age of participants with the erosion index (Table 1); there was however no correlation between the participants self-rated sensitivity and erosion index. It was also noted that participants with less than 10 years (*n* = 12) as a professional wine taster had significantly less erosion (1.53 ± 0.74) than those with over 10 years (*n* = 13) of experience (2.31 ± 0.90). Self-rated sensitivity scores of wine tasters with less than 10 years (2.82 ± 1.0) of wine tasting experience were slightly greater than those with over 10 years experience (2.06 ± 1.1); this difference was however not statistically significant.

**Figure 1:**
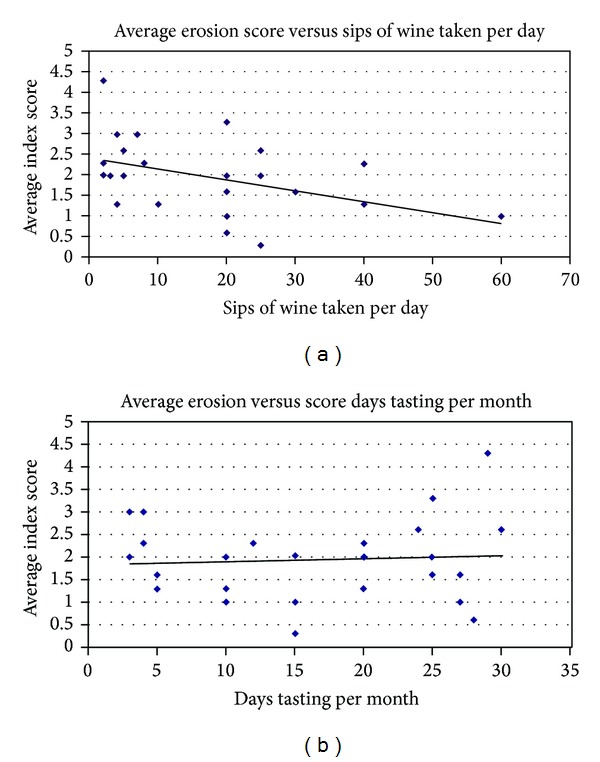
Scatter graph of dental erosion and number of wine sips. A line of best fit of the average erosion index score against the number of tasting sips per day (a) or number of days per months (b) indicates a nonlinear relationship.

The number of tasting days per month ranged from 3 to 30 days with an average of 16 days per month. The average number of sips per wine tasting per day was 18 sips, and the median number of sips was 20 per day. The highest number of sips was 60 and the lowest was 2 sips per tasting day. A scatter graph with a line of best fit of the average erosion index score against the number of tasting sips per day or number of days per months indicates a nonlinear relationship (Figures 1(a) and 1(b)). This indicates that the degree of dental erosion seen amongst professional wine tasters depends directly on the length of exposure rather than on the actual sips per day.

## 4. Discussion

A master of wine is said to taste over one hundred wines in an intensive two-and-a-half-hour period and regularly more than two hundred per week as a result of which the erosive effect can be quite extreme [18]. In the current study, involving professional wine tasters in an upcoming wine belt region in Queensland, it was noted that the average number of sips per wine tasting day was only 18 sips, and the median number of sips was 20 per day. The highest number of sips was 60 and the lowest was 2 sips per tasting day.

This study showed an increase of a significant correlation in dental erosion with the number of years spent in wine tasting (Table 1). This finding was similar to studies done by Wiktorsson et al. and Mandel who reported that the severity of tooth erosion increased with years spent as a wine taster [11, 19]. The present study also considered a significant correlation between the age of the participant and tooth erosion from wine tasting. The results showed a linear relationship between the age of wine tasters and the amount of erosion present, with the erosion scores increasing with the age of the participant. This finding may be due to a combination of factors, which may include natural tooth wear process with age [20] and a direct effect of the number of years as a professional wine taster. Tooth hypersensitivity is a concern within the wine industry [2, 19]; the present study seemed to demonstrate that wine tasters did experience some level of tooth sensitivity; however, self-rated sensitivity and erosive index of participants had both low statistical significance and a low correlation coefficient, thus making results inconclusive. This lack of correlation of the recorded erosion scores with both the number of sips per day and the degree of dentine sensitivity could be due to other factors such as age, reparative dentine deposition, plaque, brushing habits, and the use of fluorides and preventive techniques [21–23].

Mok et al. reported that factors such as acid type and concentration in addition to the pH of wine may affect the erosive potential of wine [13]. White wines had higher erosive potential than red wines, with white wine having a significantly lower pH [24, 25]. The current study had just one wine taster exclusively tasting white wine as opposed to 12 (48%) participants who tasted red wine only with another 12 (48%) who tasted both red and white wines. This study showed a small trend for higher erosion in participants tasting both red and white wines compared to those tasting only red wine; however, the results were not statistically significant. It could hence be hypothesised that the professional white wine tasters may be at a larger risk than those tasting only red wine.

One of the key variables assessed in this study was sensitivity; this study assessed self-perceived sensitivity and did not assess sensitivity clinically as the offsite nature of the study and the reluctance for a full oral examination of the participants made it difficult to assess sensitivity clinically and accurately. Sensitivity due to additional factors other than erosion could have been excluded; however, the self-perceived sensitivity indicated that the majority of the participants did not have a concern with dentine sensitivity and hence extraneous causes of sensitivity may have not played a great deal in their perception.

The accessibility of dental services is a significant problem in Australian rural communities and this presents an oral health concern for the communities under investigation [26]. It is important that these communities are made aware of the occupational hazard of wine tasting. An ideal method to prevent the detrimental effects of professional wine tasting may include a combination of frequent oral health checks and providing of adequate oral health care information and one-on-one counselling. As part of this study, all participants were provided with information on methods to prevent or decrease the detrimental effects of wine tasting. This information included asking participants to sip water immediately after the tasting of wine to aid the buffering capacity of the saliva and hence reduce the acidic period. In the present study none of the participants brushed immediately before or after wine tasting; they were encouraged to continue to do so and also informed of the advantages of using a soft-bristle brush and the need to avoid brushing just prior to or after wine tasting as plaque accumulation acts to protect and prevent enamel erosion [22]. This study showed a relatively poor understanding amongst participants of the effects of wine tasting on teeth. There also seemed to be a low uptake of additional methods of maintaining oral health. All participants in this study were informed of the role of topical fluorides, toothpaste, daily fluoride rinses, and remineralising gels in preventing and controlling the detrimental effects of professional wine tasting.

## 5. Conclusion

The study provided an insight into the erosive effect of wine amongst professional wine tasters specific to the region under study. The limited number of professional wine tasters in this upcoming wine belt of Australia makes it difficult to make recommendations for all wine tasters in Australia; however, the study does highlight the lack of dentinal hypersensitivity amongst participating professional wine tasters. This lack of dentinal hypersensitivity despite the observation of dental erosion amongst the participants may be a reason for not requesting early advice. It is hence important for the oral health practitioners to understand this occupational oral health hazard and provide early counselling and necessary invasive or noninvasive care.

## Figures and Tables

**Table 1:** The correlation coefficient (rs) and significance (*P < 0.05, ns: not significant) between dental erosion and self-rated sensitivity, age, years of wine tasting, and wine sips per day and month.

	Years of wine tasting	Age of participant	Sips per day	Days tasting per month	Self-rated sensitivity
Dental erosion score index	0.42rs*	0.43rs*	−0.02rs^ns^	−0.05rs^ns^	−0.02rs^ns^

## References

[1] Cheung A, Zid Z, Hunt D, McIntyre J (2005). The potential for dental plaque to protect against erosion using an in vivo-in vitro model: a pilot study. *Australian Dental Journal*.

[2] Gray A, Ferguson MM, Wall JG (1998). Wine tasting and dental erosion. Case report. *Australian Dental Journal*.

[3] Wiegand A, Attin T (2007). Occupational dental erosion from exposure to acids: a review. *Occupational Medicine*.

[4] Addy M (2006). Tooth brushing, tooth wear and dentine hypersensitivity—are they associated?. *Journal of the Irish Dental Association*.

[5] http://www.wineinstitute.org/files/PerCapitaWineConsumptionCountries.pdf.

[6] http://www.wineaustralia.com/Australia/LinkClick.aspx?fileticket=jUowz4QA25w%3D&tabid=235.

[7] Wine Australia (2011). *Wine Export Approval Report*.

[8] Willershausen B, Callaway A, Azrak B, Kloss C, Schulz-Dobrick B (2009). Prolonged in vitro exposure to white wines enhances the erosive damage on human permanent teeth compared with red wines. *Nutrition Research*.

[9] Conforti NJ, Cordero RE, Liebman J (2003). An investigation into the effect of three months’ clinical wear on toothbrush efficacy: results from two independent studies. *Journal of Clinical Dentistry*.

[10] http://www.scenicrim.qld.gov.au/about-the-scenic-rim.

[11] Mandel L (2005). Dental erosion due to wine consumption. *Journal of the American Dental Association*.

[12] Lussi A, Jaeggi T, Lussi A (2006). Occupation and sports. *Dental Erosion: From Diagnosis to Therapy*.

[13] Mok TB, McIntyre J, Hunt D (2001). Dental erosion: in vitro model of wine assessor’s erosion. *Australian Dental Journal*.

[14] Lussi A (2006). Erosive tooth wear: a multifactorial condition of growing concern and increasing knowledge. *Monographs in Oral Science*.

[15] Lussi A, Hellwig E, Zero D, Jaeggi T (2006). Erosive tooth wear: diagnosis, risk factors and prevention. *American Journal of Dentistry*.

[16] Buzalaf MA, Hannas AR, Kato MT (2012). Saliva and dental erosion. *Journal of Applied Oral Science*.

[17] Hooper SM, Meredith N, Jagger DC (2004). The development of a new index for measurement of incisal/occlusal tooth wear. *Journal of Oral Rehabilitation*.

[18] Chaudhry SI, Harris JL, Challacombe SJ (1997). Dental erosion in a wine merchant: an occupational hazard?. *British Dental Journal*.

[19] Wiktorsson A-M, Zimmerman M, Angmar-Månsson B (1997). Erosive tooth wear: prevalence and severity in Swedish winetasters. *European Journal of Oral Sciences*.

[20] Bartlett D, Dugmore C (2008). Pathological or physiological erosion—is there a relationship to age?. *Clinical Oral Investigations*.

[21] Murray PE, About I, Lumley PJ, Franquin J-C, Remusat M, Smith AJ (2000). Human odontoblast cell numbers after dental injury. *Journal of Dentistry*.

[22] Fischer C, Wennberg A, Fischer RG, Attström R (1991). Clinical evaluation of pulp and dentine sensitivity after supragingival and subgingival scaling. *Endodontics & Dental Traumatology*.

[23] Smales R, Yip K, Yip K, Smales R, Kaidonis J (2006). Prevention and control of tooth erosion. *Tooth Erosion: Prevention and Treatment*.

[24] Brand HS, Tjoe Fat GM, Veerman ECI (2009). The effects of saliva on the erosive potential of three different wines. *Australian Dental Journal*.

[25] Willershausen B, Callaway A, Azrak B, Kloss C, Schulz-Dobrick B (2009). Prolonged in vitro exposure to white wines enhances the erosive damage on human permanent teeth compared with red wines. *Nutrition Research*.

[26] Steele L, Pacza T, Tennant M (2000). Rural and remote oral health, problems and models for improvement: a Western Australian perspective. *The Australian Journal of Rural Health*.

